# Study of anti-*Toxoplasma gondii* effect of mPEG–PCL copolymeric loaded with pyrimethamine, in vitro

**DOI:** 10.1186/s13568-025-01876-8

**Published:** 2025-05-02

**Authors:** Mobina Gholami, Hossein Danafer, Mitra Sadeghi, Ahmad Daryani, Seyed Abdollah Hosseini, Shirzad Gholami

**Affiliations:** 1 Zanjan Pharmaceutical Nanotechnology Research Center, Zanjan University of Medical Sciences, Zanjan, Iran; 2 Department of Medicinal Chemistry, School of Pharmacy, Zanjan University of Medical Sciences, Zanjan, Iran; 3 Student Research Committee, Mazandaran University of Medical Sciences, Sari, Iran; 4 Toxoplasmosis Research Center, Communicable Disease Institute, Mazandaran University of Medical Sciences, Sari, Mazandaran Iran; 5 Department of Parasitology and Mycology, School of Medicine, Mazandaran University of Medical Science, 18 Km of Khazar Abad Road, Sari, Iran

**Keywords:** Antiparasitic effect, mPEG–PCL copolymer nanoparticles, Pyrimethamine, *Toxoplasma gondii*, In vitro

## Abstract

The discovery of new drugs with minimal toxicity is crucially important in the search for effective treatments for toxoplasmosis. The study aimed to assess the effectiveness of pyrimethamine-loaded mPEG–PCL copolymeric nanoparticles (mPEG–PCL–Pyr) against *Toxoplasma gondii* (*T. gondii*) tachyzoites. The nanoparticles were fabricated using a nano-precipitation method and characterized using DLS and AFM. Through MTT assay, the anti-toxoplasmic activity was gauged. Healthy and infected cell viability, infection index, proliferation index, plaque count, and size were evaluated. Results indicated a maximum drug loading capacity of 11.23% in the nanoparticles. Toxicity testing on Vero cells infected with tachyzoites revealed notable cytotoxic effects at pyrimethamine-equivalent concentrations of 20 and 40 mg/mL in the mPEG–PCL copolymeric nanoparticle formulation. However, as the results indicated, the administration of mPEG–PCL–Pyr resulted in the reduction of intracellular proliferation, infection index, and the number and size of plaques in comparison to uninfected treated cells (*P* < 0.05). The synthesized Nano drug exhibited slightly better performance than pyrimethamine, despite the lack of statistical significance in the observed distinction (*p* > 0.05). The study demonstrated that mPEG–PCL copolymeric nanoparticles containing pyrimethamine effectively impede the *in vitro* replication of *T. gondii*. The study demonstrated that mPEG–PCL copolymeric nanoparticles containing pyrimethamine effectively impede the in vitro replication of *T. gondii*. The impact of pyrimethamine resembled that of the synthesized nano-drug on *T. gondii* tachyzoites, suggesting the potential of these nanoparticles as a promising avenue for toxoplasmosis treatment.

**Supplementary Information:**

The online version contains supplementary material available at 10.1186/s13568-025-01876-8.

## Introduction

Toxoplasmosis is an intracellular protozoan infection caused by *Toxoplasma gondii* (*T. gondii*) that has the ability to infect most warm-blooded vertebrates. Toxoplasmosis is due to the transmission of the parasite through the consumption of water and food contaminated to the oocytes and the parasite cysts of the toxoplasma parasite (Elmore et al. 2010; Hill et al. 2005).

In individuals with healthy immune systems, *T. gondii* infection is typically asymptomatic or causes only mild symptoms. However, in infants infected during fetal development and immunocompromised individuals, the infection can lead to severe complications and even death (Hohlfeld et al. 1989; Hosseini et al. 2019; Sarvi et al. 2019).

Various drugs such as pyrimethamine, spiramycin, and clindamycin are used for the treatment of toxoplasmosis. Currently, the standard treatment for toxoplasmosis involves a combination of pyrimethamine and a sulfonamide (Alday and Doggett 2017; Derouin 2001). However, this treatment has posed challenges for clinicians, particularly in individuals with severe immune disorders such as AIDS patients. About 50% of HIV positive patients treated with a combination of pyrimethamine and sulfadiazine experience severe toxicity, which can result in the discontinuation of treatment (Chirgwin et al. 2002). The treatment of toxoplasmosis is challenging due to the toxic effects of available drugs, and reinfection can occur rapidly. Furthermore, there is no drug currently available that can completely eliminate the parasite. Some drugs only have the ability to restrict the growth of the parasite during the active reproduction phase. Furthermore, the chemotherapy presently advised demonstrates efficacy solely against the tachyzoite stage of the parasite and does not have any influence on cysts (Antczak et al. 2016; Montazeri et al. 2018, 2017). Given the existing challenges, the development and production of new anti -*Toxoplasma* drugs to prevent and reduce the harmful effects of these parasites on the host cells will be a promising alternative. An approach provided by WHO includes the use of natural ingredients as well as the use of nanotechnology in the treatment of parasitic diseases. This strategy may provide a safer and more effective tool for the treatment of toxoplasmosis while minimizing negative side effects (Assolini et al. 2017; Jafarpour et al. 2021).

Amphiphilic fragmented copolymers are one of the most important synthesis systems used to create self-assembled nanostructures (Perin et al. 2021). Biodegradable polycaprolactone polymers (PCL) are one of the common polymers used in the production of nanoparticles. It is less toxic and more stable than liposomes and other drug delivery systems, making it an ideal candidate for drug delivery applications (Łukasiewicz et al. 2021; Mandal and Shunmugam 2020; Obisesan et al. 2023). Although PCL has many advantages due to its lipophilic properties, such as ease of formulation and rapid removal of nanoparticles containing this polymer by the body's reticuloendothelial system, the use of hydrophilic polymers, such as mPEG–PCL copolymer, is gaining more attention in nanoparticle synthesis (Danafar 2016; Peng et al. 2015).

In this study, the anti-parasitic effects of mPEG–PCL copolymer nanoparticles loaded with pyrimethamine (mPEG–PCL–Pyr) were investigated on the tachyzoite of *T. gondii* strain RH *in vitro*. To the best of our knowledge, this is the first study to evaluate the anti-parasitic potential of these nanoparticles against *T. gondii.*

## Materials and methods

### Ethical considerations

This *in vitro* experimental study was conducted in accordance with the ethical standards established by the Zanjan University of Medical Sciences ethics committee, as outlined in their approved organizational guidelines. The ethics committee assigned a unique ethics number to this project (Code of Ethics: IR.ZUMS.REC.1400.099).

### Materials

mPEG having a molecular weight of 5000 Da was procured from Aldrich located in St. Louis, USA (CAS.81323). ε-caprolactone with a purity of 98% was bought from Acros based in New Jersey, USA (CAS.502443). Pyrimethamine was procured from Sigma-Aldrich in the USA. Stannous 2-ethylhexanoate (Sn(Oct)2) was acquired from Aldrich in St. Louis, USA (CAS.301100). All remaining chemicals and solvents were of laboratory-grade quality and were procured locally, then used as received.

#### Synthesis of mPEG–PCL copolymer

This investigation involved the production of mPEG–PCL copolymers using anhydrous caprolactone monomer, dry polyethylene glycol as the initiator molecule, and tin octanate catalyst through ring-opening polymerization. The method was employed to synthesize the mPEG copolymer PCL with a 1:4 ratio of mPEG to CL.

#### Binary copolymer analysis mPEG–PCL

To verify the creation of mPEG–PCL copolymers and nanoparticles, as well as to ascertain their vibrational characteristics, FT-IR spectroscopic analysis was conducted within the scope of this investigation. The FT-IR spectra of various samples were obtained using a Bruker Tensor 27 device. Additionally, the structure of the mPEG–PCL binary copolymer was determined using H-NMR spectroscopy, which was conducted at 25 °C using a Bruker 400 MHz device. Deuterated chloroform (CDCl3) was used as the solvent, while trimethylsilane (TMS) was utilized as an internal standard for the H-NMR spectroscopic analysis (Soheilian et al. 2005).

#### Preparation of nanoparticles loaded with pyrimethamine from mPEG–PCL copolymers

In this investigation, the nano-precipitation method was utilized for the preparation of nanoparticles. In this method, 6 mg of pyrimethamine and 20 mg of copolymer were dissolved in 2 mL of acetone. The resulting mixture was added to 20 mL of deionized water and stirred vigorously using a magnetic stirrer for 2–3 h at room temperature until the organic solvent evaporated as much as possible. The suspension of nanoparticles was then collected by centrifugation at 15,000 rpm and the supernatant was discarded. The remaining material was transferred to a container and dried using a freezer to prepare a suspension containing nanoparticles. The solvent was evaporated, and a dry powder was obtained without any residual solvent (Soheilian et al. 2005; Ramazani et al. 2018).

### Characterization of the mPEG–PCL ***copolymers***

#### Nanoparticle measurement

To determine the particle size distribution of the prepared micelles, dynamic light scattering (DLS) was employed in this study. The measurements were conducted using a Malvern Instruments Nano ZS model located in Worcestershire, UK (Chu et al. 2016).

#### Determining the morphology of nanoparticles by AFM

The morphology of the nanoparticles was analyzed using atomic force microscopy (AFM) in this investigation. AFM measurements were conducted using a Nano Wizard 2 model from JPK in Berlin, Germany. For sample preparation, the micelles were diluted with water, and a droplet of 2 μL was deposited onto a freshly cleaved mica substrate measuring 1 cm^2^. The sample was then allowed to air-dry before being analyzed using intermittent contact mode in AFM (Manjili et al.^2016^).

### Measurement of pyrimethamine using spectrophotometry

#### Preparation of calibration curve from samples

To create a standard curve for the measurement of pyrimethamine concentration, ultraviolet light spectroscopy was utilized in this study. To generate the calibration curve, a solution with a concentration of 1 mg/mL of pyrimethamine was initially prepared. Subsequently, solutions with varying concentrations were derived from the initial solution, and the absorbance of each concentration was measured three times using a spectrophotometer at a wavelength of 480 nm. Finally, the calibration curve was constructed by plotting the obtained concentrations against their respective absorbance values.

#### Drug loading efficiency in nanoparticles

To determine the amount of drug loaded in the nanoparticles, 1 mg of completely dry nanoparticles was dissolved in 1 mL of water. The absorbance of this solution was measured at a wavelength of 480 nm using a UV spectrophotometer to determine the concentration of pyrimethamine. The amount of drug in 1 mg of nanoparticles was then calculated based on the calibration curve of pyrimethamine and the dilution factor of the solution. The total amount of encapsulated drug in the entire precipitate was also measured. Finally, using the following formula, the loading efficiency of pyrimethamine in the nanoparticles was calculated (Gan et al. 2017):

$$ \begin{gathered} {\text{Drug}}{\mkern 1mu} {\text{loading}}{\mkern 1mu} {\text{content}}\left( \% \right) \hfill \\ = \frac{{{\text{Weight}}{\mkern 1mu} {\text{of}}{\mkern 1mu} {\text{the}}{\mkern 1mu} {\text{drug}}{\mkern 1mu} {\text{in}}{\mkern 1mu} {\text{nanoparticles}}}}{{{\text{Weight}}{\mkern 1mu} {\text{of}}{\mkern 1mu} {\text{the}}{\mkern 1mu} {\text{nanoparticles}}}} \hfill \\ \end{gathered} $$

#### Host cells and the strain of the parasite

Within the context of this research, *in vitro* experiments were carried out employing Vero cells obtained from the renal fibroblast of the African green monkey (ATCC No. CCL-81). The Vero cell line was cultured in RPMI-1640 medium containing 10% heat-inactivated fetal bovine serum (FBS), along with 100 μg/mL of penicillin and streptomycin obtained from Sigma, USA. The cells were incubated at a temperature of 37 °C in an environment with 5% carbon dioxide.

The *T. gondii* RH strain was sourced from the Toxoplasmosis Research Center situated at Mazandaran University of Medical Sciences, Sari, Iran. Tachyzoites of *T. gondii* were sustained via intraperitoneal transfers within female BALB/c mice at the Toxoplasmosis Research Center, Mazandaran University of Medical Science, Sari, Iran. At intervals of 3–4 days, tachyzoites of *T. gondii* were retrieved from the peritoneal cavities of the infected mice. (Montazeri et al. 2016).

#### Experimental design

In this study, Vero cells were exposed to different treatments. The groups studied included: I) mPEG–PCL nanoparticles, II) mPEG–PCL–Pyr, III) Pyrimethamine (positive control), and IV) RPMI (negative control). These treatments were used at different concentrations.

#### Cytotoxicity assay of mPEG–PCL copolymer nanoparticles loaded with pyrimethamine to Vero cells

Viability assessment was conducted using Vero cells, with 2 × 10^4^ cells per well in 96-well plates, with each well containing 200 μL of RPMI medium supplemented with 10% FBS. The plates were then kept at 37 °C with 5% CO_2_ for 24 h to allow the cells to adhere and grow. Afterward, the Vero cells were exposed to different treatments for 24 h: mPEG–PCL nanoparticles, mPEG–PCL–Pyr, Pyrimethamine (alone). These treatments were applied at various concentrations: 5, 10, 20, and 40 mg/mL and each concentration was tested in triplicate. Furthermore, for the purpose of evaluating the potential cytotoxicity of Dimethyl sulfoxide (DMSO) on Vero cells, an additional set of cells was subjected to treatment with 0.25% DMSO in RPMI 1640 medium.

The control cell group was exposed to RPMI 1640 medium enriched with 10% FBS. Subsequently, on the next day, the viability of Vero cells was gauged through a tetrazolium salt (MTT) colorimetric assay. The optical density (OD) of the formazan product was assessed by measuring its absorbance at a wavelength of 570 nm through a spectrophotometer.Utilizing the acquired data, the 50% cytotoxic concentrations (CC_50s_) were computed employing GraphPad Prism 6.0 software developed by GraphPad Software, Inc., situated in San Diego, USA (Sadeghi et al. 2022; Teimouri et al. 2018).

#### Impact of mPEG–PCL copolymer nanoparticles loaded with pyrimethamine on the invasion and intracellular multiplication of Toxoplasma gondii during the post-treatment phase

Vero cells were grown on 13-mm circular glass slides positioned within 24-well plates, with each well containing 2 × 10^4^ cells in 200 μL of medium. The plates were then incubated for 72 h in RPMI medium supplemented with 10% FBS, at a temperature of 37°C and with a 5% CO_2_ environment. Following the incubation duration, the cells were exposed to *T. gondii* infection at a ratio of 10:1 parasite per cell. Following 3 h of incubation to allow the infection to occur, the cells were treated with the following substances: Pyrimethamine at a concentration of 40.76 mg/mL, mPEG–PCL nanoparticles at a concentration of 69.95 mg/mL and mPEG–PCL–Pyr at a concentration of 24.88 mg/mL. Moreover, control cells were subjected to infection without any subsequent treatment.

After an additional 24-h incubation, the glass slides with attached Vero cells were delicately rinsed using cold PBS to eliminate any loose cells or debris. Then, the cells were immobilized in a 10% buffered formalin solution for 24 h to ensure the effective preservation of cellular structures. In the final step of the process, the coverslips with fixed cells were stained using a 1% toluidine blue solution for a brief period of 10 s. Subsequently, the stained coverslips were analyzed using a light microscope (Nikon, Japan) to assess both the *T. gondii* infection index (which refers to the count of infected cells among every 100 observed cells) and the proliferation of parasites within the cells (indicated by the total count of tachyzoites per 100 examined cells) (Barbosa et al. 2012).

#### Determining the viability of *Toxoplasma gondii* tachyzoites using trypan blue exclusion method

In the pre-treatment phase, the viability of *T. gondii* tachyzoites that underwent treatment was evaluated using the Trypan blue exclusion assay. This assay allows researchers to determine the mortality of the parasites. To determine the optimal timing for direct exposure of the parasites to the test compounds, *T. gondii* tachyzoites (2 × 10^5^) were subjected to treatment with pyrimethamine (40.76 mg/mL), mPEG–PCL (69.95 mg/mL), and mPEG–PCL–Pyr (24.88 mg/mL) for two distinct time intervals: 30 min and 24 h. After these specific exposure times, the viability of the treated tachyzoites was evaluated using the Trypan blue exclusion assay. This analysis aids in identifying the ideal timeframe for directly exposing the parasites to the test compounds, in order to achieve the intended outcomes. Following Trypan blue staining, the *T. gondii* tachyzoites were quantified using a Neubauer chamber under a light microscope. The control group comprised tachyzoites treated with RPMI-1640 medium. Viable parasites were distinguished by their translucent cytoplasm and enumerated using an optical microscope. On the other hand, nonviable parasites, which took up the blue dye, were also counted. To compute the percentage of viable cells, the subsequent formula was employed (Strober 1997):

$$ \begin{gathered} {\text{Viable cells }}\left( \% \right) \hfill \\ = \left( {{\text{total viable cells}}/{\text{total cells }}\left[ {{\text{viable }} + {\text{ dead}}} \right]} \right) \times 100 \hfill \\ \end{gathered} $$

#### Impact of novel quinolone derivatives on the invasion and intracellular multiplication of Toxoplasma gondii during the pre-treatment phase

The influence of mPEG–PCL, mPEG–PCL–Pyr, and pyrimethamine on the invasion and proliferation of *T. gondii* within Vero cells was evaluated using experimental infection models with a pre-treatment design. For this investigation, *T. gondii* tachyzoites (2 × 10^5^) were exposed to pre-treatment with distinct concentrations of pyrimethamine (40.76 mg/mL), mPEG–PCL (69.95 mg/mL), and mPEG–PCL–Pyr (24.88 mg/mL) over a 30-min interval. As a negative control, tachyzoites treated with RPMI were employed.

Afterward, the treated tachyzoites were introduced to Vero cells cultivated on 13-mm circular glass slides positioned within 24-well plates. Each well contained 2 × 10^4^ cells in 200 μL of medium. The plates were subsequently placed in an incubator set at 37°C with a 5% CO_2_ atmosphere for a duration of 24 h. After the 24-h incubation period, the glass slides with adherent cells were carefully washed using cold PBS to remove any non-adherent cells or debris. Following the incubation period, the cells were fixed by immersing them in a 10% buffered formalin solution for 24 h to ensure the preservation of their cellular structures. In the next step, the fixed cells on the glass slides were stained with 1% toluidine blue for a short duration of 10 s. This staining process aids in the enhancement of cell visualization and the identification of any intracellular parasites. Subsequent to staining the cells with toluidine blue, they were scrutinized and quantified utilizing a light microscope to assess both the infection index and the intracellular proliferation of *T. gondii* (Barbosa et al. 2012).

#### Impact of novel quinolone derivatives on the plaque assay of *Toxoplasma gondii*

To investigate the influence of treatment on the size and quantity of plaques formed in the growth medium, Vero cells were cultivated within 24-well plates. The culture medium employed was RPMI 1640 supplemented with 10% fetal bovine serum (FBS), and the plates were kept at a temperature of 37°C with a 5% CO_2_ environment. Each well contained 2 × 10^4^ cells in 1000 μL of the medium. The cells were allowed to grow and adhere in this culture condition for 72 h.

After the initial 72-h incubation period, The Vero cells were subjected to infection with *T. gondii* tachyzoites of the RH strain. In each well, 2 × 10^5^ tachyzoites were added in 200 μL of RPMI 1640 medium supplemented with 10% FBS. After 3 h, the cells were washed with medium to remove any extracellular parasites, and then they were treated with pyrimethamine (40.76 mg/mL), mPEG–PCL (69.95 mg/mL), and mPEG–PCL–Pyr (24.88 mg/mL) at their respective concentrations. Control cells were also infected with *T. gondii* tachyzoites but were left untreated. After 5 days of incubation, the cells were washed with cold PBS to remove any debris or non-adherent cells. The cells were then fixed with a 10% buffered formalin solution for 24 h to preserve their cellular structures. Under a light microscope, the stained Vero cells were meticulously observed to ascertain the count and dimensions of the generated plaques (Montazeri et al. 2016).

### Statistical analysis

The data were subjected to analysis using GraphPad Prism software, version 8.4.2 (GraphPad Software, Inc., San Diego, CA, USA). The comparison between the control and test groups (both positive and negative) was conducted utilizing the One-Way ANOVA followed by the Tukey test for post hoc analysis. A p-value less than 0.05 was considered statistically significant.

## Results

### Synthesis and characterization of mPEG–PCL copolymer

A diblock copolymer composed of monomethoxypoly (ethylene glycol) (mPEG) and poly(ε-caprolactone) (PCL) was effectively synthesized using the ring-opening polymerization of ε-caprolactone. The hydroxyl end group of mPEG was employed as the initiator for the ring-opening process. The formation of copolymer micelles and the nanostructures of these micelles were confirmed using AFM following preparation and identification. The size of nanoparticles loaded with SF (presumably referring to a drug or compound) was determined using the dynamic light scattering method. The mean hydrodynamic diameter (Z) of the mPEG–PCL micelles loaded with SF was found to be 118 nm, and the zeta potential (a measure of the surface charge) was measured to be 12 mV. Additionally, the polydispersity index (PDI), which indicates the distribution of particle sizes in the sample, was determined to be 0.122.

### Investigation of FT-IR spectroscopic spectrum of mPEG–PCL copolymer nanoparticles

In this research, the Fourier Transform Infrared (FT-IR) spectrum of the mPEG–PCL copolymer was examined and analyzed. The absorption band observed at the wavenumber of 1108 cm^1^ corresponds to the stretching of bonds involving carbon and oxygen (C–O). The sharp and intense absorption band observed at 1729 cm^1^ indicates the presence of carbonyl ester groups (C=O), providing evidence of the formation of the mPEG–PCL copolymer. Furthermore, at the wavenumber of 2948 cm^1^, the stretching vibrations of methylene hydrogens (–CH_2_–) are observed, which is characteristic of the C–H absorption. Additionally, the absorption in the region around 3415 cm^1^ is related to the hydroxyl group (–OH), indicating the presence of this functional group in the copolymer structure (Supplementary 1).

### Investigating the H-NMR spectrum of mPEG–PCL copolymer nanoparticles

**Fig. 1:**
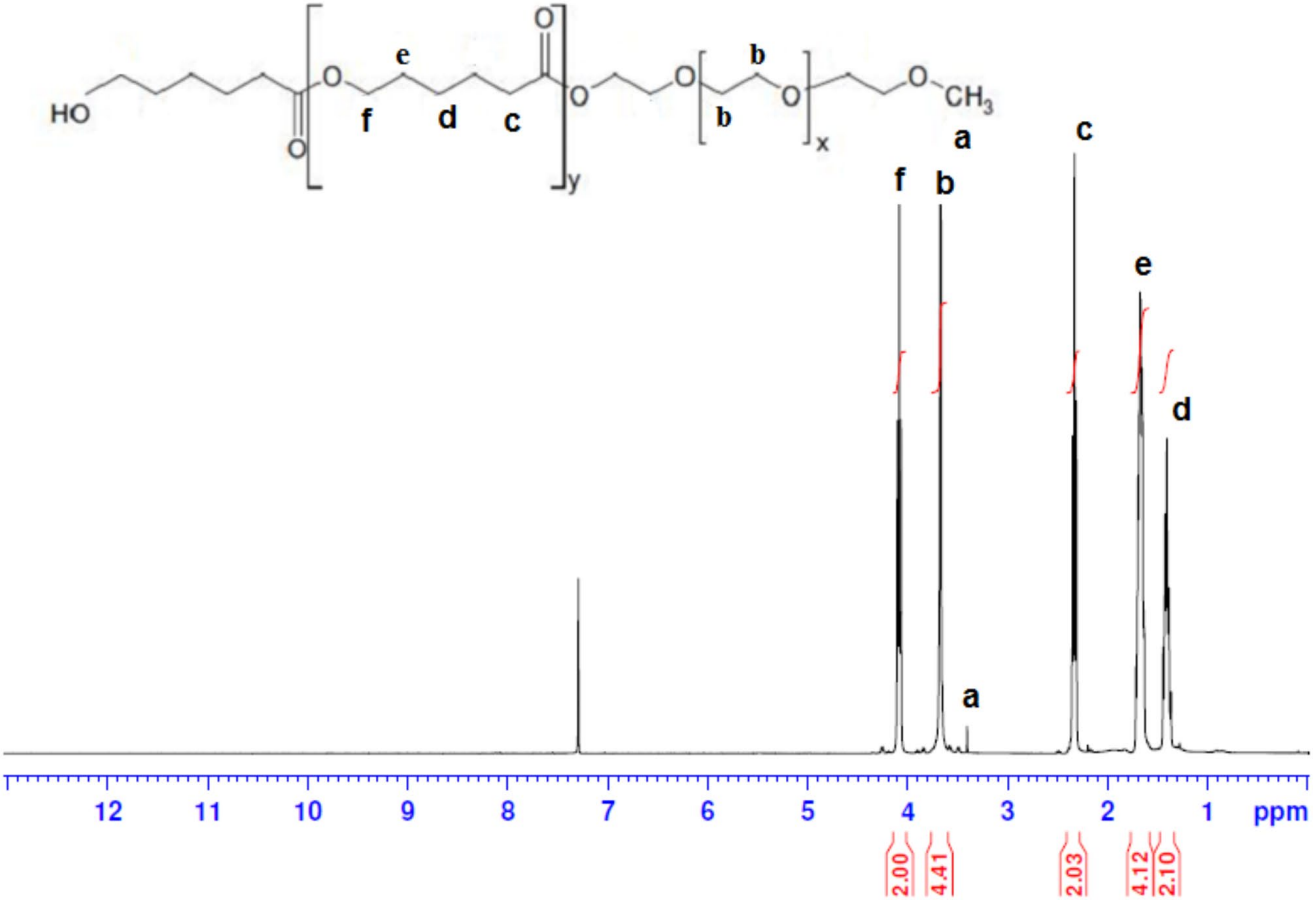
mPEG–PCL copolymer and H NMR spectrum of mPEG–PCL diblock copolymer in CDCl3

The structure and composition of the synthesized mPEG-PCL copolymer were elucidated through H-NMR spectroscopy, carried out in a CDCl3 solvent. The H-NMR spectrum provides valuable information about the different hydrogen environments present in the copolymer, allowing for the identification of specific molecular groups. Here is a breakdown of the observed peaks (Fig. 1):

Peaks corresponding to the hydrogens of the methoxy group and the methyl groups of mPEG.

- Peak at 393.3 ppm (3H, a): This peak corresponds to the hydrogens of the methoxy (CH_3_O–) group in mPEG.
- Peak at 658.3 ppm (4H, b): This peak corresponds to the hydrogens of the methyl groups (–CH_3_) in mPEG.

Peaks corresponding to the hydrogens of caprolactone in PCL:

- Peak at 432.1 ppm (2H, d): This peak corresponds to the methylene (–CH2–) hydrogens in the caprolactone unit.
- Peak at 657.1 ppm (2H, e): This peak corresponds to the methine (–CH–) hydrogens in the caprolactone unit.
- Peak at 332.2 ppm (2H, c): This peak corresponds to the methylene (–CH2–) hydrogens in the caprolactone unit.
- Peak at 072.4 ppm (2H, f): This peak corresponds to the methine (–CH–) hydrogens in the caprolactone unit.

### Measuring the dimensions and zeta potential of mPEG–PCL copolymer nanoparticles containing pyrimethamine using DLS apparatus

**Fig. 2:**
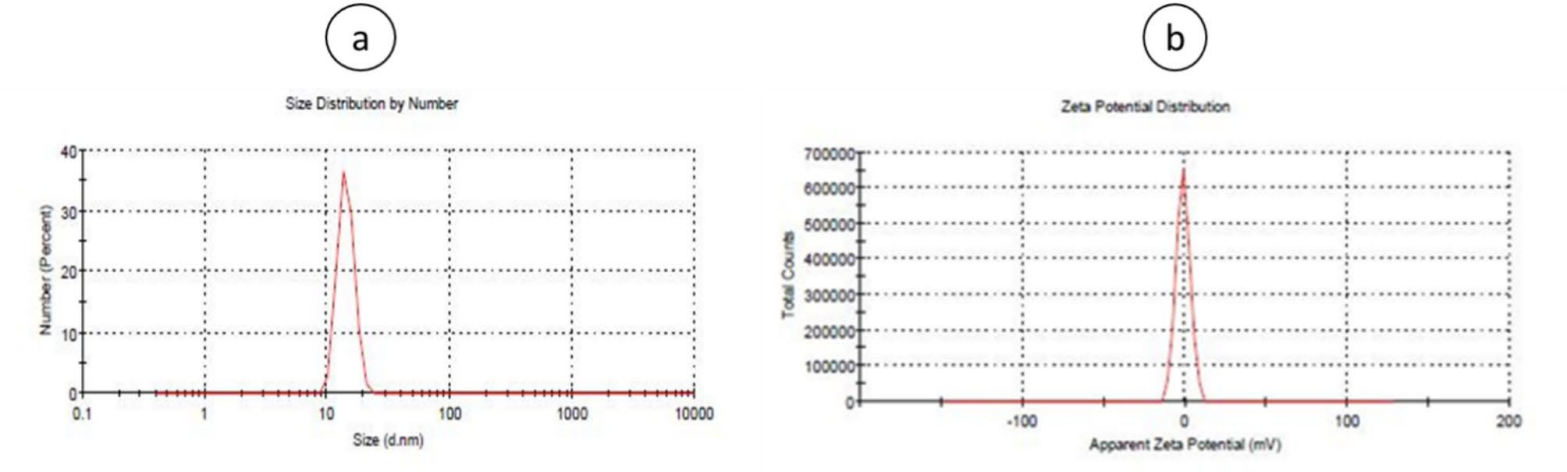
Particle size distribution (**a**) and zeta potential **b** of mPEG–PCL–pyrimethamine copolymer

The size spectrum of the prepared polymersome mPEG–PCL nanoparticles, as determined by DLS, was found to be approximately 261 nm. This measurement indicates the hydrodynamic diameter of the nanoparticles, which includes both the particle core and the surrounding solvation layer. The zeta potential of the mPEG–PCL copolymer, when loaded with the pyrimethamine drug, was determined to be − 0.98 mV using DLS (Fig. 2).

### Analyzing of FT-IR spectra of mPEG–PCL copolymer nanoparticles incorporated with pyrimethamine

In the FT-IR spectra of the mPEG–PCL copolymer, the absorption band at 2948 cm^1^ corresponds to the stretching of C–H bonds. Similarly, the absorption peak at 1108 cm^1^ is indicative of the stretching of C–O bonds, while the absorption at 1729 cm^1^ is associated with the C=O stretching within the carbonyl group. In the FT-IR spectrum related to pyrimethamine drug, the peak area of 1600–1400 cm^1^ is related to C=C and C=N of the aromatic ring. The peak observed in the 3149 cm^1^ region corresponds to C–H of the aromatic ring, which overlaps with the peaks in the FT-IR spectrum of the mPEG–PCL copolymer nanoparticles loaded with pyrimethamine. The peak area 3310 and 3467 cm^1^ corresponds to H–N–H of NH_2_. From the comparison of pyrimethamine drug spectrum data with mPEG–PCL copolymer nanoparticle spectrum data, it is clear that pyrimethamine-related regions have been observed in mPEG–PCL copolymer nanoparticles loaded with pyrimethamine, which is a proof of pyrimethamine loading in the nanoparticle (Supplementary 2).

### Investigating the morphology of mPEG–PCL copolymer nanoparticles loaded with pyrimethamine drug

**Fig. 3:**
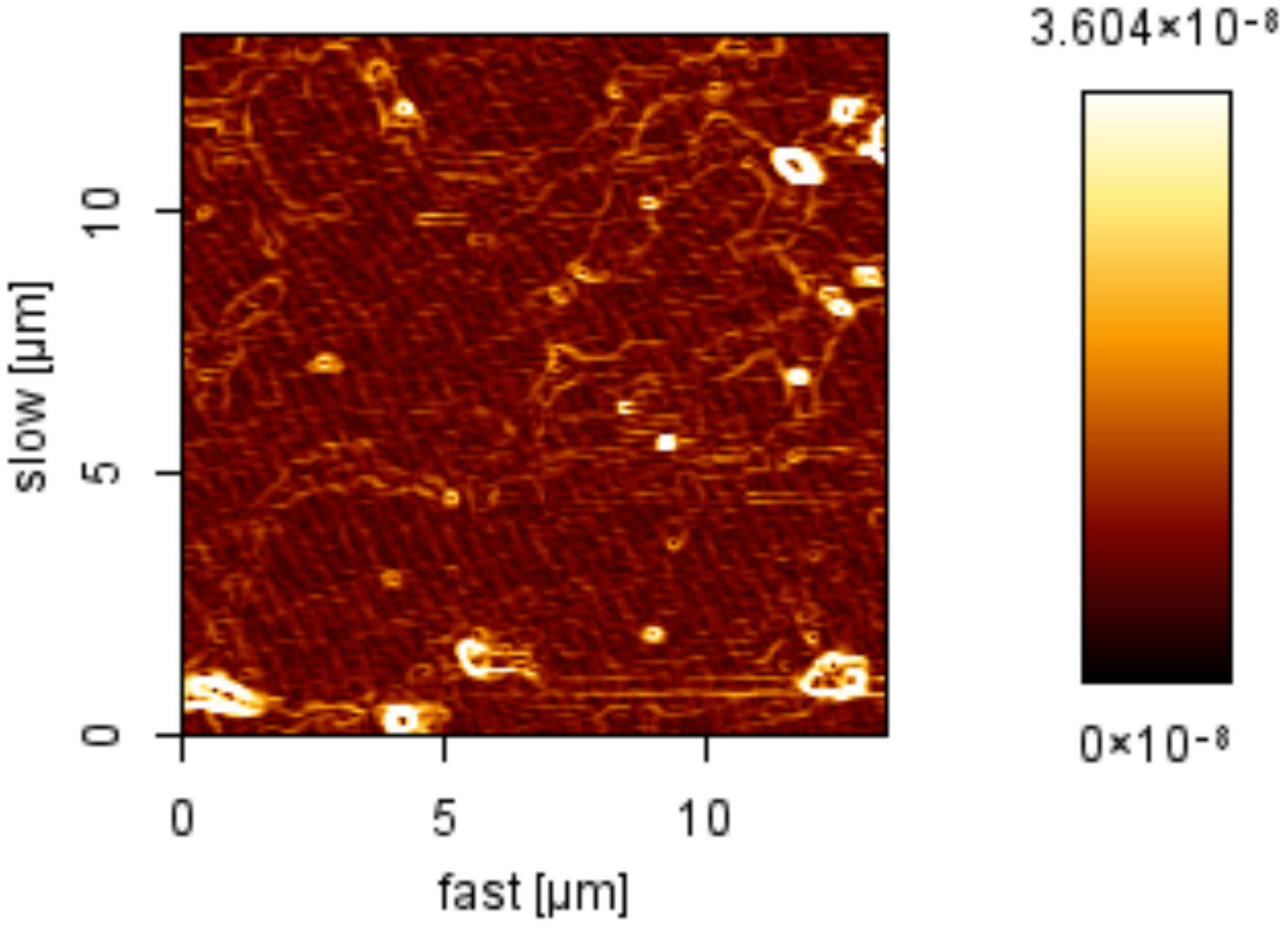
AFM image of drug-loaded nanoparticle

Using an AFM device, the morphology of the nanoparticles was examined and its image is shown in Fig. 3. Based on the provided image, the average dimensions of the nanoparticles were calculated to be approximately 150 nm.

### Pyrimethamine calibration curve

**Fig. 4:**
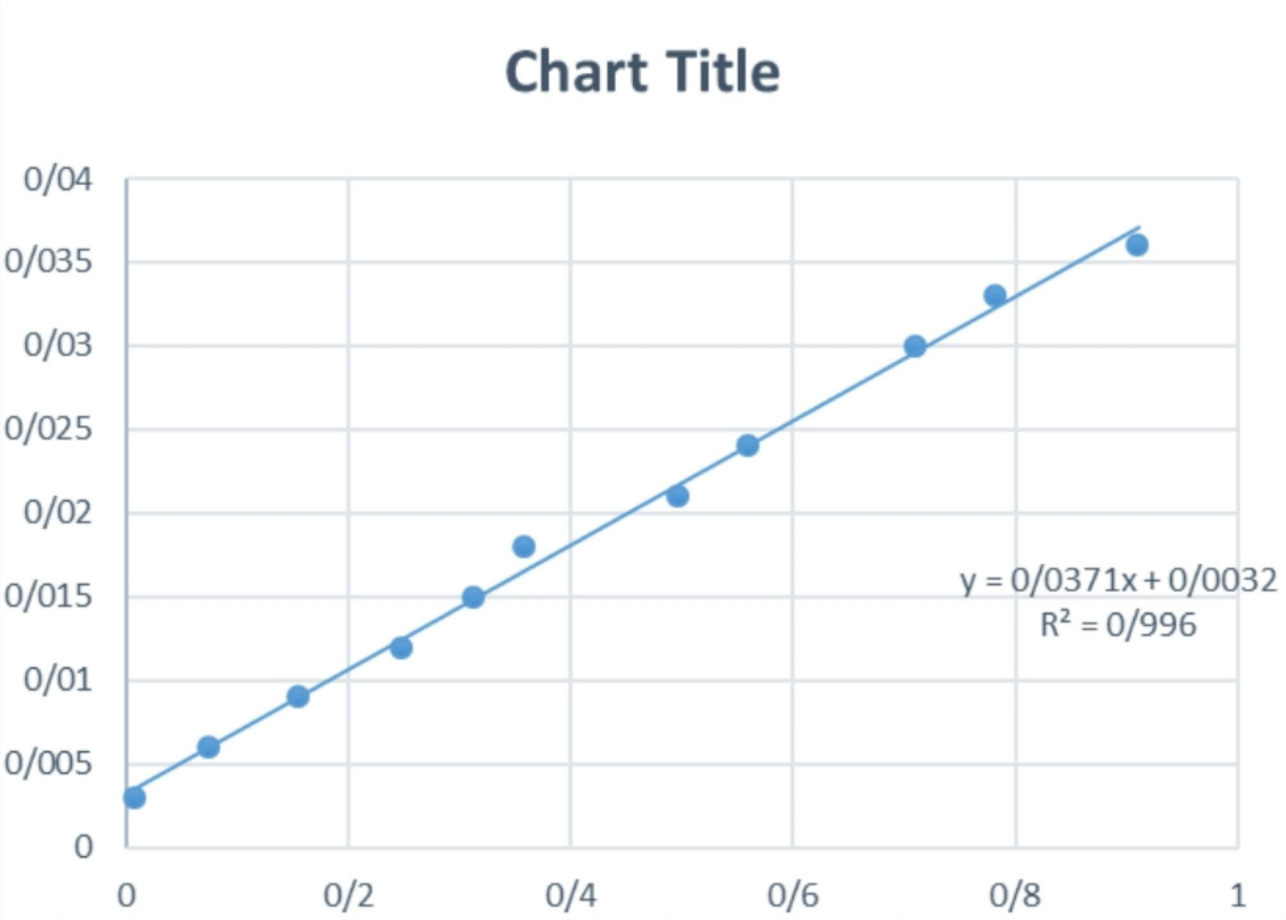
Pyrimethamine Calibration Curve at 480 nm Wavelength

Table 1 Absorbance data of standard samples, the accuracy and precision of spectrophotometry and Fig. 4 shows the pyrimethamine calibration curve obtained at 480 nm wavelength. By using the linear equation obtained from this graph, pyrimethamine concentration estimation and drug release analysis have been done.

**Table 1:** The accuracy and precision of the spectrophotometric method for the drug pyrimethamine

Concentration (mg/mL)	RSD intraday (%)	RSD interlay (%)	Accuracy (%)
0.003	4.37	3.98	102 ± 0.31
0.018	8.13	7.56	98 ± 0.89
0.036	6.51	5.36	0.64 ± 101

### The sensitivity of the spectrophotometric method

The measurable limit of the device (LOD) and the detectable limit of the device (LOQ) are important parameters that provide information about the sensitivity of the spectrophotometric method used for pyrimethamine analysis. These limits help determine the lowest concentration of pyrimethamine that can be reliably measured and detected by the spectrophotometer. The limit of detection (LOD) refers to the minimum substance concentration that can be accurately differentiated from the inherent measurement background noise. In other words, it represents the minimal pyrimethamine concentration that can be identified with a certain level of confidence using the spectrophotometric method, although precise quantification might not be assured, the LOD is determined to be 0.003 mg/mL, meaning that pyrimethamine concentrations below this value may not be reliably distinguishable from the background noise. The Limit of Quantification (LOQ) is the lowest concentration of a substance that can be reliably measured and quantified with acceptable precision and accuracy. It is a step further from the LOD and indicates the lowest concentration at which accurate quantitative measurements can be made. For this spectrophotometric method, the LOQ is estimated to be 0.002 mg/mL, meaning that pyrimethamine concentrations above this value can be reliably measured and quantified with acceptable accuracy and precision.

### Examining the amount of drug loading in the copolymer

The amount of pyrimethamine drug loading in nanoparticles was calculated. The results derived from this investigation suggest that the maximum drug loading capacity of the nanoparticles was 11.23%.

### Drug release study in vitro conditions in buffer with pH = 7.4

The study of pyrimethamine drug release from mPEG–PCL copolymer nanoparticles was conducted in PBS buffer environment with pH 7.4 in a specific time period and the results show the percentage of pyrimethamine released from nanoparticles at different time points.

The maximum release of pyrimethamine occurred at 96 h and approximately 70% of the loaded pyrimethamine was released from the nanoparticles by this time.

This drug release profile is crucial in understanding the release kinetics and behavior of pyrimethamine from the mPEG–PCL nanoparticles. The release rate and duration can significantly impact the therapeutic efficacy and dosage regimen of the drug delivery system. By monitoring the drug release over time, researchers can optimize the nanoparticle formulation to achieve the desired drug release profile for specific medical applications (Supplementary 3).

### Assessment of cell viability in vitro

**Fig. 5:**
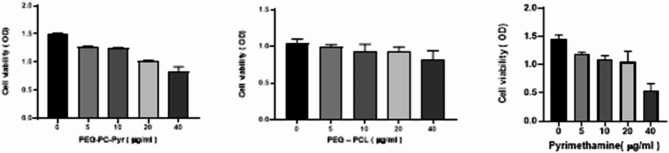
Cellular viability determined by MTT assay in Vero cells treated with mPEG–PCL copolymer, mPEG–PCL–Pyr and pyrimethamine in several concentrations ranging from 5 to 40 μg/mL. The obtained data from experiments in triplicates are presented as mean ± SEM. Significant differences in relation to untreated cells (control) (*P* < 0.05)

The cytotoxicity of mPEG–PCL, mPEG–PCL–Pyr, pyrimethamine, and DMSO was analyzed in Vero cells using the MTT assay. Table 2 and Fig. 5 presents cellular viability in Vero cells treated with mPEG–PCL copolymer, mPEG–PCL–Pyr and pyrimethamine. According to the results presented in Table 2, administering pyrimethamine alone led to a significant decrease in Vero cell viability across all tested concentrations (5, 10, 20, and 40 mg/mL) in comparison to untreated cells. Conversely, both mPEG–PCL copolymer and mPEG–PCL–Pyr were less cytotoxic than pyrimethamine.

**Table 2:** In vitro biological data of mPEG–PCL copolymer, mPEG–PCL–Pyr

Drug (mg/mL)	CC_50_ (mg/mL)	IC_50_ (mg/mL)	SI*
mPEG–PCL	306	69.95	4.37
mPEG–PCL–Pyr	141.7	24.88	5.695
Pyrimethamine	385.9	40.76	9.47

IC_50a_ = Concentration required to reduce Vero cell growth by 50% (μg/ml)

CC_50b_ = Concentration required to inhibit *T. gondii* by 50% (μg/ml)

SIc = CC_50_/IC_50_ = Selectivity index

Based on the results obtained from the graphs and the determination of the IC_50_, the concentrations of 69.95, 24.88 and 2.75 (mg/mL) for mPEG–PCL copolymer, mPEG–PCL–Pyr and pyrimethamine have been selected for further investigation.

### In vitro assay to assess invasion and proliferation at the post-treatment stage

**Fig. 6:**
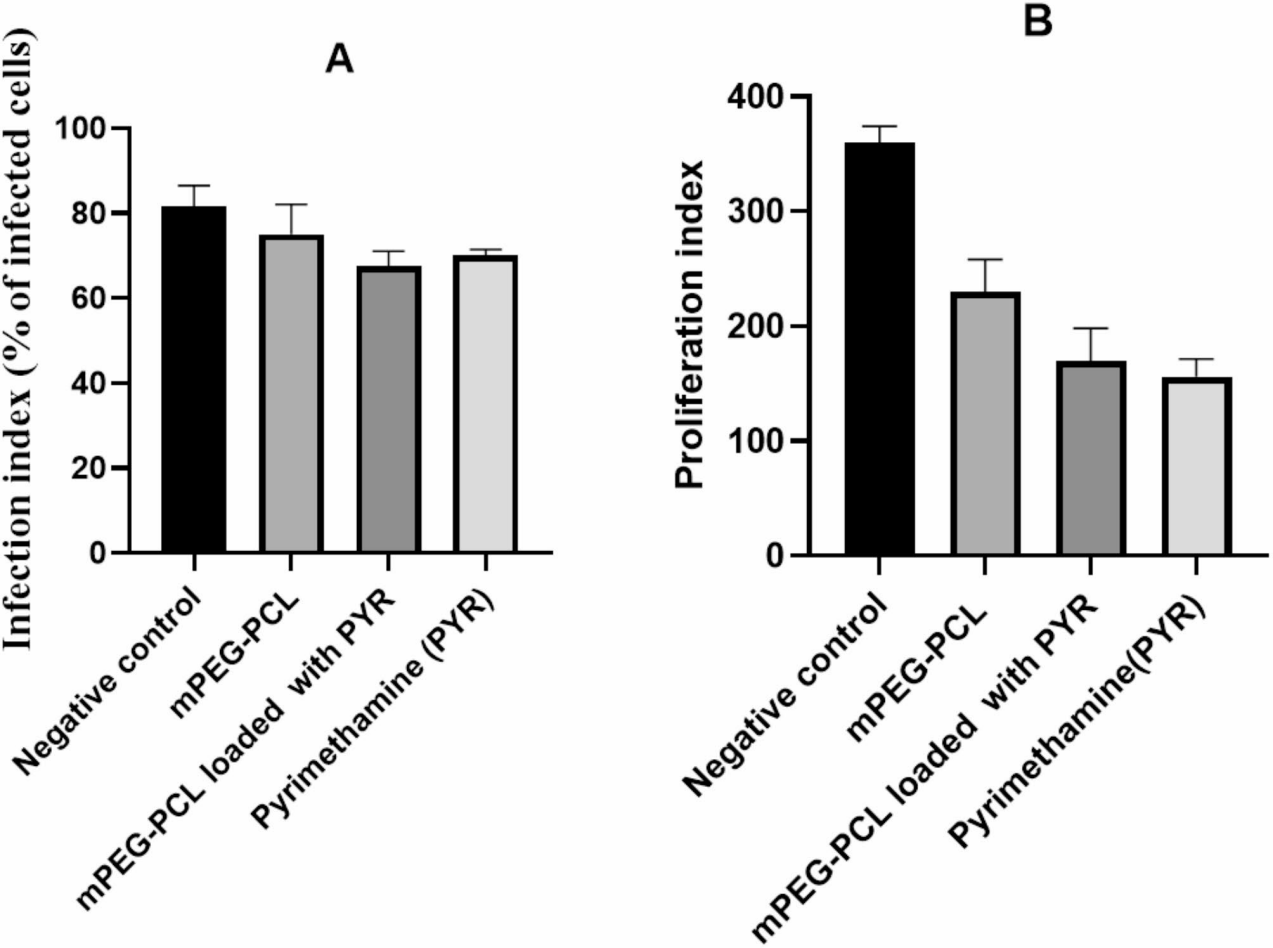
Infection index (% of infected cells) (**A**) and parasite proliferation (total number of tachyzoites per 100 cells) **B** in the Vero cells treated mPEG–PCL, mPEG–PCL–Pyr and pyrimethamine at the concentration of 69.95, 24.88 and 40.76 mg/mL determined by the blue toluidine staining. The obtained data from two independents experiments in triplicates are presented as mean ± SEM. No significant differences were observed regarding their reduction in proliferation index (*P* > 0.05)

To determine the dose–response effect on the infection index and intracellular proliferation of parasite, the experimentation with Vero cell culture and infection with *T. gondii* was performed with various concentrations of mPEG–PCL copolymer, mPEG–PCL–Pyr, and pyrimethamine (69.95, 24.88, and 40.76 mg/mL, respectively) in comparison to untreated parasites as negative controls (Fig. 6).

As illustrated in Fig. 6, treatment with mPEG–PCL copolymer, mPEG–PCL–Pyr, pyrimethamine drug resulted in reduction in both infection index (75 ± 7.07, 67.5 ± 3.53, and 70 ± 1.41, respectively) and intracellular proliferation (230 ± 28.28, 170 ± 28.28, and 156 ± 15.56 respectively), compared to the untreated cells (81.5 ± 4.95 and 360 ± 14.14, respectively). Moreover, administering pyrimethamine (whether as a standalone drug or as part of the Nano medicine) led to a noteworthy reduction in both the infection rate and the overall count of tachyzoites per 100 cells, in comparison to the infected cells that were left untreated. However, no significant differences were observed among the mPEG–PCL, mPEG–PCL–Pyr, and pyrimethamine investigated regarding their reduction in proliferation index (*P* > 0.05).

### Viability of tachyzoites

This method was designed to promptly gauge the impact of these substances on *T. gondii*, given that the parasite is an obligate intracellular protozoan with limited viability outside the host cell. To assess the effectiveness of pyrimethamine (40.76 mg/mL), mPEG–PCL (69.95 mg/mL), and mPEG–PCL–Pyr (24.88 mg/mL) on *T. gondii*, two different time points were considered: 30 min and 24 h.

In the 24-h test, we found that mPEG–PCL, mPEG–PCL–Pyr, and pyrimethamine for tachyzoites were cytotoxic and reduced the survival of tachyzoites. While, when parasites were directly exposed to mPEG–PCL, mPEG–PCL–Pyr, and pyrimethamine in 30 min, it was observed that the tachyzoites achieved maximum survival within this relatively short timeframe. Specifically, the survival rates of the parasites after 30 min were 99%, 99%, and 97%, respectively, compared to the negative control group (100%). Therefore, for the pre-treatment test, considering that direct exposure of the parasites to mPEG–PCL, mPEG–PCL–Pyr, and pyrimethamine resulted in maximum viability at 30 min, this time frame was chosen to continue the work (Supplementary 4).

### Evaluation of invasion and proliferation prior to the commencement of treatment

**Fig. 7:**
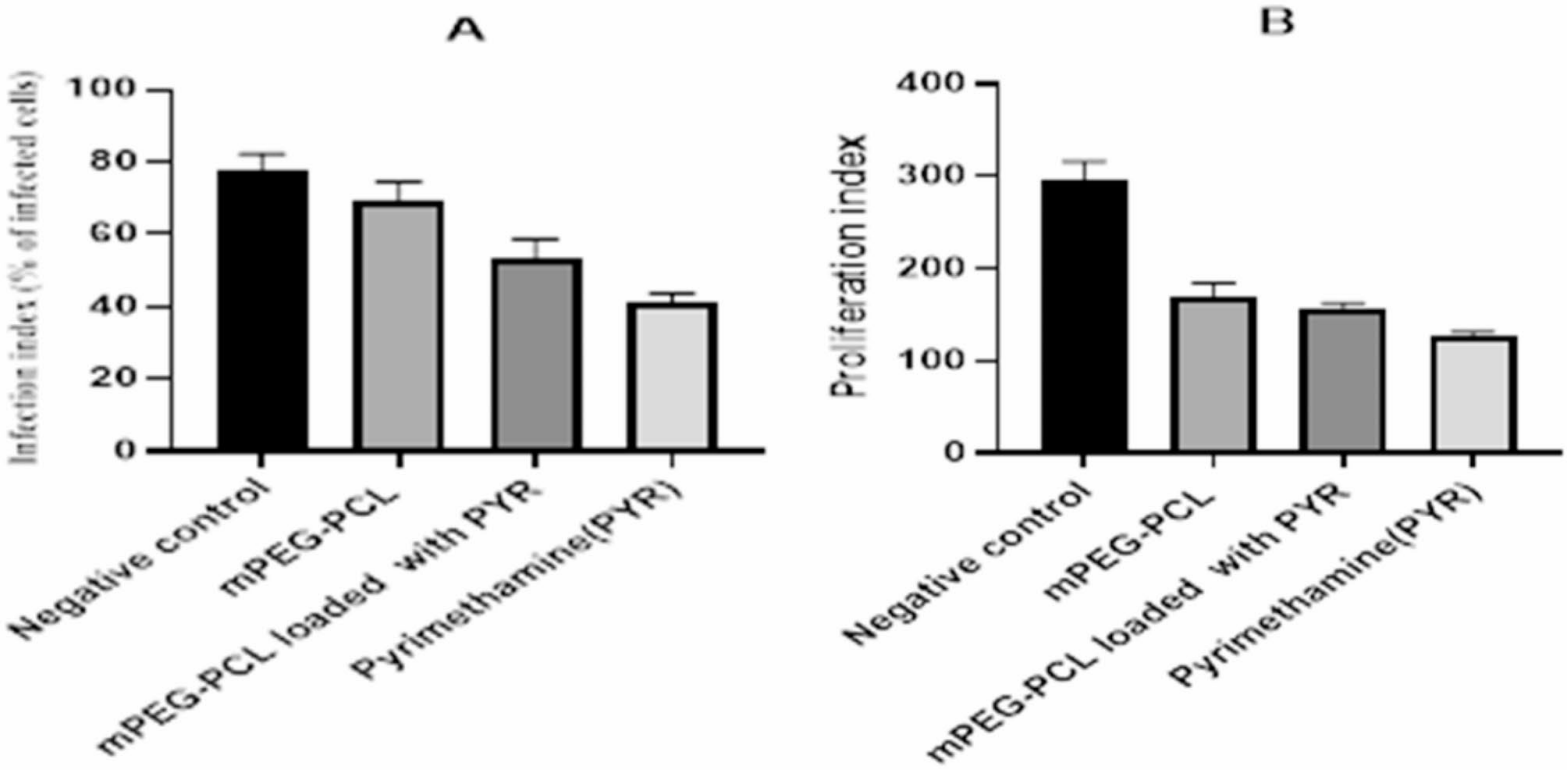
Infection index (% of infected cells) (**a**) and parasite proliferation **b** in Vero cells treated with mPEG–PCL, mPEG–PCL–Pyr and pyrimethamine at the concentration of 69.95, 24.88 and 40.76 mg/mL determined by the blue toluidine staining (Control cells were infected, but not treated). The obtained data from two independents experiments in triplicates are presented as mean ± SEM. No significant differences were observed regarding their reduction in proliferation index (*P* > 0.05)

According to the information depicted in Fig. 7, the application of mPEG–PCL, mPEG–PCL–Pyr, and pyrimethamine for treatment led to a considerable and statistically significant decrease in the infection index (69 ± 5.66, 53 ± 5.66, and 41 ± 2.83, respectively). In comparison, the infection index value for the negative control group was 78 ± 4.24. The findings indicated that there was no notable distinction between mPEG–PCL–Pyr and pyrimethamine regarding their impact on reducing the infection index (*P* > 0.05). This indicates that both mPEG–PCL–Pyr and pyrimethamine had a similar impact in reducing the rate of *T. gondii* infection in the Vero cell culture. Conversely, a significant and statistically noteworthy reduction in the intracellular proliferation index was evident for all three treatments: mPEG–PCL, mPEG–PCL–Pyr, and pyrimethamine (170 ± 14.14, 157 ± 5.66, and 127.50 ± 3.53, respectively; *P* < 0.05), in contrast to untreated cells (295 ± 21.21). No substantial distinction was noted among the examined mPEG–PCL, mPEG–PCL–Pyr, and pyrimethamine concerning their reduction in the proliferation index (*P* > 0.05).

### Plaque assay 

**Fig. 8:**
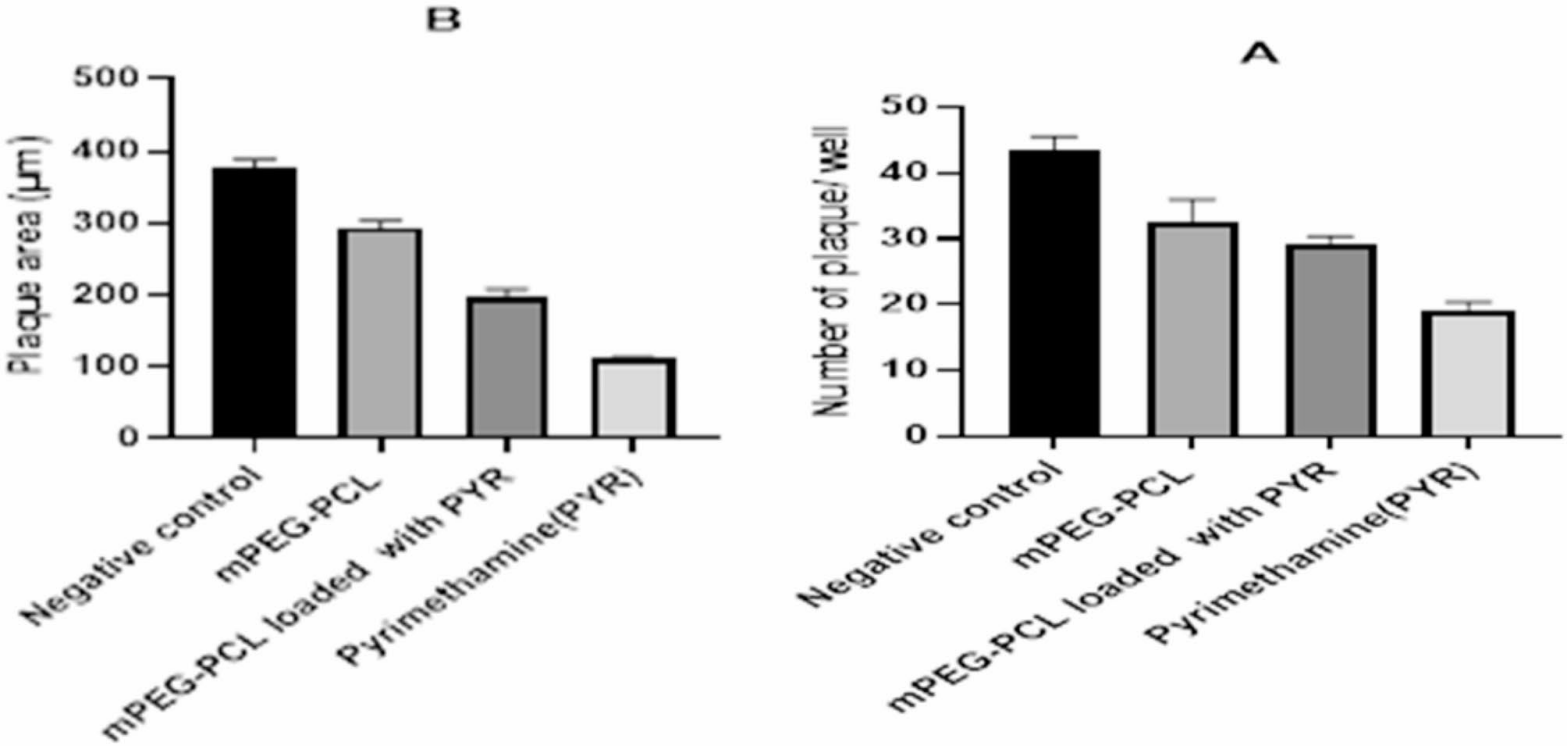
Number (**a**) and area of plaques **b** in the Vero cells treated with mPEG–PCL, mPEG–PCL–Pyr and pyrimethamine at the concentration of 69.95, 24.88 and 40.76 mg/mL determined by the blue toluidine staining (Control cells were infected, but not treated). The obtained data from two independents experiments in triplicates are presented as mean ± SEM (*P* < 0.05). Significant differences were observed regarding their reduction in proliferation index (*P* < 0.05)

Plaque assay with the scale bar has been shown in Fig. 8. Treatment with mPEG–PCL, mPEG–PCL–Pyr, and pyrimethamine led to a significant reduction in the number of plaques formed in the infected Vero cells (31.3 ± 3.21, 27.67 ± 2.52, and 17.67 ± 2.52, respectively) Compared to the cells that were not treated (41.33 ± 4.04; *P* < 0.05; Fig. 8). Also, statistical analysis showed significant difference in a number of plaques between derivatives and pyrimethamine/ciprofloxacin (P > 0.05).

Moreover, the addition of mPEG–PCL, mPEG–PCL–Pyr, and pyrimethamine to the infected Vero cells resulted in a notable decrease in the size of the plaques (298.33 ± 12.58, 187.33 ± 18.56, and 107.67 ± 5.13 μm, respectively) compared to the untreated cells (387.33 ± 20.60 μm; *P* < 0.05; Fig. 8).

## Discussion

Toxoplasmosis is attributed to *T. gondii*, an intracellular protozoan responsible for a prevalent infection in both humans and animals. The spread of this infection is widespread across the globe (Attias et al. 2020). Current treatment options include drugs like pyrimethamine, sulfonamide, spiramycin, clindamycin, and tetracycline. However, these drugs have limited effectiveness in eliminating parasites and can cause adverse effects, especially in those with health conditions or weak immune systems (Alday and Doggett 2017; Neville et al. 2015). Therefore, Researchers are focused on developing new drugs with fewer side effects to improve toxoplasmosis treatment.

Over the last decade, significant progress has been achieved in drug delivery systems, with nano drug delivery being particularly noteworthy in cancer treatment. Nano drug delivery systems have shown promise in improving drug efficacy and targeting. Amphiphilic block copolymers are utilized to create self-assembled nanostructures like micelles, liposomes, or nanoparticles, which can encapsulate drugs and protect them during transportation in the body, facilitating targeted delivery to specific sites. This targeted drug delivery reduces damage to healthy tissues and enhances drug accumulation at diseased sites, leading to increased treatment effectiveness and fewer side effects. Nano drugs offer several advantages over traditional drugs, including reduced side effects, the ability to administer smaller doses, improved drug delivery, and lower drug toxicity (Benavides et al. 2023; Danafar et al. 2014; Danafar et al. 2016). In this study focusing on treating toxoplasmosis caused by *T. gondii*, we are investigated mPEG–PCL–Pyr for their antiparasitic effect on *T. gondii* tachyzoite in vitro. By leveraging nano drug delivery systems, we aim to harness nanotechnology’s advantages to enhance toxoplasmosis treatment and potentially pave the way for more efficient and safer therapeutic approaches in the future.

In this study, cell viability assessments were conducted using the MTT method to evaluate the effects of different treatments. The interventions comprised the solitary application of mPEG–PCL copolymer, mPEG–PCL–Pyr, and the pyrimethamine pharmaceutical agent. These interventions were contrasted with an untreated control cohort. The results indicated that all three treatments led to a decrease in cell viability when compared to the untreated group. This suggests that each treatment was effective in reducing the viability of the cells, indicating their potential antiparasitic activity against *T. gondii* tachyzoite. Notably, the outcomes from laboratory experiments indicated that both mPEG–PCL copolymer and mPEG–PCL–Pyr demonstrated elevated CC_50_ values within Vero cells when juxtaposed with the lone administration of the pyrimethamine medication. The higher CC_50_ values for the copolymer-based treatments imply that they were comparatively less toxic to the Vero cells at the given concentrations than the pyrimethamine drug.

KarimiPourSaryazdi et al. (2019) assessed the anti-toxoplasmic impact of silver nanoparticles produced via ginger plant extracts through *in vitro* experimentation employing the MTT technique on macrophage cells. Consistent with findings from our own investigations, the outcomes of this study revealed that ginger-synthesized silver nanoparticles exert a fatal influence on *Toxoplasma* parasites and trigger apoptosis (KarimiPourSaryazdi et al. 2019).

In line with the results of the present study, Arafa et al. (2023) showed that chitosan nanoparticles with sulfonamide-triazole hybrids (CNP) performed better than sulfadiazine in vitro, reducing *T. gondii* viability at lower concentrations and preserving host cell morphology (Arafa et al. 2023).

Also, Norouzi et al. (2024) stated in their study that CH-NP-ROS nanoparticles showed almost complete eradication of tachyzoites (98.95%) in vitro, which surpassed free rosuvastatin (76%). Also, in this study, rosuvastatin nanoparticles were able to reduce the parasite load in the peritoneal fluid and spleen, and were consistent with the effect of sulfadiazine/pyrimethamine) Norouzi et al. 2024).

In another study by Hashemi et al. (2023), spiramycin nanoparticles achieved 100% tachyzoite mortality in 120 min at 250 μg/mL, compared to 70% for the non-nano suspension (S-Spi). The nanoemulsion also showed no cytotoxicity to Vero cells at therapeutic doses) Hashemi et al. 2023).

Die Vergara-Duque et al. (2020) undertook a research endeavor aimed at examining the impact of nanosilver on the morphology of *T. gondii*. Employing both Fluorescence Microscopy and Scanning Microscopy methodologies, the researchers detected alterations in the configuration of the oocyst. Furthermore, they noted a reduction in the parasite's half-life as a result of their investigation. This study highlights the potential of nanosilver as an agent to alter the morphology and survival of *T. gondii* (Vergara-Duque et al. 2020).

In the investigation carried out by Hagras et al. (2019), the researchers investigated the successful treatment of experimental acute toxoplasmosis using spiramycin loaded in chitosan nanoparticles. They compared the effectiveness of spiramycin-metronidazole and spiramycin loaded by chitosan nanoparticles (CS NP) to the conventional treatment of *T. gondii* with spiramycin, specifically assessing their ability to penetrate tissues and the blood–brain barrier (BBB). The study found that CS NP demonstrated the highest efficiency in treating acute toxoplasmosis compared to the other treatments tested. The results highlighted that the combination of CS nanoparticles with spiramycin showed potent antiparasitic effects while maintaining a non-toxic nature. This combination not only exhibited strong efficacy in combating the infection but also displayed enhanced penetration into tissues and the BBB. Based on these findings, the researchers concluded that chitosan nanoparticles loaded with spiramycin hold great potential as a promising agent for the treatment of human toxoplasmosis (Hagras et al. 2019).

The results of post-treatment effects with mPEG–PCL copolymer, mPEG–PCL–Pyr and pyrimethamine drug (concentrations of 69.95, 24.88 and 2.75 μg/mL, respectively) on parasite invasion and proliferation showed that mPEG–PCL copolymer and the mPEG–PCL–Pyr have performed almost the same as pyrimethamine. Although the effect of mPEG–PCL–Pyr on parasite proliferation index was greater than pyrimethamine. In this experiment, mPEG–PCL–Pyr was better than mPEG–PCL copolymer.

In addition, in this study, the results of pre-treatment with mPEG–PCL copolymer, mPEG–PCL–Pyr and pyrimethamine drug (concentrations of 69.95, 24.88 and 2.75 μg/mL, respectively) on parasite invasion and proliferation showed that mPEG–PCL copolymer and mPEG–PCL–Pyr acted almost the same as pyrimethamine and led to a significant reduction in parasite invasion and proliferation compared to the untreated control group.

The findings of the investigation suggest that both the mPEG–PCL copolymer and mPEG–PCL–Pyr demonstrate comparable effectiveness to the conventional pyrimethamine in terms of diminishing parasite invasion and proliferation. This suggests that these nanoparticle formulations could serve as viable alternatives for the treatment of parasitic infections. Notably, the mPEG–PCL–Pyr showed a higher impact on the parasite proliferation index compared to standard pyrimethamine, indicating its potential superiority in inhibiting parasite growth.

Similarly, in the research conducted by Farhadi et al. (2018), the researchers investigated the effectiveness of mPEG–PCL nanoparticles loaded with flubendazole as a potential treatment against protoscolexes and cysts of *Echinococcus granulosus*. The results demonstrated that the nanoparticles loaded with flubendazole exhibited higher efficacy compared to free flubendazole in both in vitro and in vivo. This suggests that the nanoparticle formulation enhanced the therapeutic effects of flubendazole against the parasite (Farhadi et al. 2018).

Algmi et al. (2019) carried out an investigation titled “Anti-toxoplasmic Activity of Silver Nanoparticles Synthesized with *Phoenix dactylifera* and *Ziziphus spina-christi*.” Their findings demonstrated that the aforementioned synthesized silver nanoparticles effectively mitigated liver damage induced by toxoplasmosis through the inhibition of enzyme activities. Based on their findings, the researchers concluded that silver nanoparticles produced with plant extracts hold potential in reducing toxoplasmosis-related liver damage by targeting enzyme activities (Alajmi et al. 2019).

Nazareth Costa et al. (2021) investigated the effects of biogenic silver nanoparticles on the control of *T. gondii* infection in both human trophoblast cells and explants. In this study, it was concluded that AgNp-Bio can reduce *T. gondii* infection in trophoblast cells and villous explants. Therefore, Nazareth Costa et al.’s study reveals that biogenic silver nanoparticles can reduce *T. gondii* infection in human trophoblast cells and villous explants, indicating the capacity of silver nanoparticles to hinder parasite proliferation (Costa et al. 2021). Like this study, in our research, the ability of mPEG–PCL–Pyr to reduce the proliferation of *T. gondii* was observed. Indeed, the parallel observation in the present study, where mPEG–PCL–Pyr demonstrated an ability to reduce the proliferation of *T. gondii*, aligns with the positive outcomes reported by others and this consistency in results across different studies highlights the robustness and potential utility of nanoparticle-based therapies in addressing parasitic infections.

Taji et al. (2022) research demonstrates the anti-parasitic effect of silver nanoparticles, chitosan, and curcumin against *Blastocyst*, which causes giardiasis. The positive results obtained in laboratory conditions highlight the potential efficacy of silver nanoparticles as a treatment option for giardiasis, a common intestinal parasitic infection. The combination of silver nanoparticles with chitosan and curcumin may offer enhanced anti-parasitic effects, making it a promising approach for further investigation (Taji et al. 2022).

Similarly, Mohammed et al. (2019) study investigates the antibacterial effect of silver nanoparticles on *Leishmania tropica*, another parasitic organism causing leishmaniasis. The results indicate that silver nanoparticles effectively inhibit the growth of both *Leishmania* promastigotes and amastigotes, which are different life stages of the parasite. Furthermore, the study reveals that the inhibitory effect of silver nanoparticles is potentiated when combined with ultraviolet radiation. This finding suggests that silver nanoparticles could be a valuable option for treating leishmaniasis and highlights the potential of combining nanoparticle treatments with other modalities to improve efficacy (Mohammed et al. 2019). Taken together with the results of other studies mentioned earlier, the evidence consistently points to the effectiveness of silver nanoparticles against a range of parasitic infections. The anti-parasitic activity of silver nanoparticles, including their ability to inhibit parasite growth and proliferation, makes them attractive candidates for further exploration as potential therapeutic agents.

Plaque assays are commonly used to measure the antiviral or antiparasitic activity of compounds, and in this study, the effect of mPEG–PCL–Pyr on the number and size of parasite-induced plaques was evaluated. The plaque assay results in the present study provide valuable evidence that mPEG–PCL–Pyr has shown similar effects to the conventional drug pyrimethamine in combating *Toxoplasma* parasites.

The study indicates that pyrimethamine alone exhibited cytotoxicity at certain concentrations, while the nanoparticle formulation demonstrated improved performance. The enhanced drug efficacy and reduced toxicity in the nanoparticle system can be attributed to several factors: Nano-encapsulation provides sustained and targeted release of pyrimethamine, reducing peak plasma concentrations that often cause toxicity. Nanoparticles also increase the solubility and stability of the drug, enhancing its efficacy at lower doses. On the other hand, nanoparticles have the potential to be designed to deliver the drug specifically to infected cells and minimize exposure to healthy tissues, thereby reducing side effects. As shown in studies, encapsulated formulations exhibit better tolerability compared to non-encapsulated forms. Collectively, these features improve the therapeutic index of pyrimethamine while overcoming its inherent cytotoxic limitations (Awashra et al. 2023; Xiong et al.^2022^).

As a conclusion, the fact that the mPEG–PCL–Pyr had a similar effect to positive control (pyrimethamine) shows that these nanoparticles effectively inhibit the proliferation of the parasite and reduce the size and number of plaques formed by the parasite. Hence, this type of compounds can be subjected to animal studies for finding new treatment of toxoplasmosis. This resemblance shows that nanoparticles -based formulations are capable of presenting pyrimethamine in a way that maintains its therapeutic effectiveness against parasites. The positive effects observed in this study show that the mPEG–PCL-copolymer nanoparticles have the potential to increase the delivery and bioavailability of the pyrimethamine and lead to the comparative effects of anti-parasite with the conventional drug. The findings of this study highlight the potential of mPEG–PCL–Pyr as a promising and effective treatment option for parasitic infections, including toxoplasmosis. By leveraging the advantages of nanoparticle-based drug delivery systems, such as improved bioavailability, targeted delivery, and reduced side effects, this approach could revolutionize antiparasitic therapies. While further validation in complex biological systems is necessary, this research lays a strong foundation for the application of nanotechnology in combating parasitic diseases.

In this study, the nanoparticles in question were only studied *in vitro*, and for more accurate results, it is recommended that the results be studied *in vivo* and drug delivery to specific tissues such as the brain, eye, etc. be investigated. It is also suggested that in future studies, new derivatives of this nanodrug synthesized in this study be used on chronic stages.

## Electronic supplementary material

Below is the link to the electronic supplementary material.

Supplementary Material 1.

## Data Availability

All data is provided in the manuscript and in additional files. All data are available on request.

## Abbreviations

Pyr: Pyrimethamine
IC_50_: Concentration required to reduce Vero cell growth by 50% (μg/mL)
CC_50_: Concentration required to inhibit *T. gondii* by 50% (μg/mL)
SIc: CC_50_/IC_50_ = Selectivity index
FBS: Fetal bovine serum
CS: Chitosan
