# Supplementary material for: Study of anti-Toxoplasma gondii effect of mPEG–PCL copolymeric loaded with pyrimethamine, in vitro
Source: AMB Express. 2025 May 2;15:69. doi: 10.1186/s13568-025-01876-8 (PMC12048374; doi:10.1186/s13568-025-01876-8)
Supplement: Supplementary file 1 — Supplementary Material 1. [file 13568_2025_1876_MOESM1_ESM.docx]

**supplementary Information**

**Study of anti-*Toxoplasma gondii* effect of mPEG - PCL copolymeric loaded with pyrimethamine, in vitro**

Mobina Gholami ^1^, Hossein Danafer ^1,^ ^2^, Mitra Sadeghi ^3, 4^, Ahmad Daryani ^4, 5^, Seyed Abdullah Hosseini ^4, 5^, Shirzad Gholami ^4, 5, *^

*^1^ Zanjan Pharmaceutical Nanotechnology Research Center, Zanjan University of Medical Sciences, Zanjan, Iran.*

*^2^* *Department of Medicinal Chemistry, School of Pharmacy, Zanjan University of Medical Sciences, Zanjan, Iran.*

*^3^ Student Research Committee, Mazandaran University of Medical Sciences, Sari, Iran*

*^4^ Toxoplasmosis Research Center, Communicable Disease Institute, Mazandaran University of Medical Sciences, Sari, Mazandaran, Iran*

*^5^ Department of Parasitology and Mycology, School of Medicine, Mazandaran University of Medical Science, Sari, Iran*

***Corresponding authors:** Dr. Shirzad Gholami

Department of Medical Parasitology and Mycology 18 Km of Khazar Abad Road, School of Medicine, Mazandaran University of Medical Sciences, Sari, Iran

**Email:** [sgholami200@gmail.com](mailto:sgholami200@gmail.com)

**Figure S1** FT-IR spectrum of mPEG-PCL copolymer.


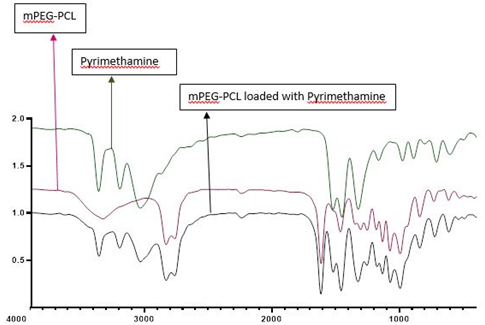


**Figure S2** FT-IR spectrum of mPEG-PCL copolymer, pyrimethamine drug and mPEG-PCL copolymer nanoparticle loaded with pyrimethamine.

**Figure S3** Release of pyrimethamine in buffer medium with pH= 7.4 in vitro

**Table S1** Mortality and Viability of *Toxoplasma gondii* tachyzoites treated with drugs using Trypan blue exclusion assay

| **Treatment** | **Tachyzoites Viability (%) 30 min** | **Tachyzoites Mortality (%) 24 h** |
| --- | --- | --- |
| Negative Control | 100 | 2 |
| mPEG-PCL | 99 | 50 |
| Nano medicine | 99 | 68 |
| Pyrimethamine | 97 | 80 |
